# Unpacking multi-level governance of antimicrobial resistance policies: the case of Guangdong, China

**DOI:** 10.1093/heapol/czac052

**Published:** 2022-07-01

**Authors:** Olivia Sinn Kay Chan, Didier Wernli, Ping Liu, Hein Min Tun, Keiji Fukuda, Wendy Lam, YongHong Xiao, Xudong Zhou, Karen A Grépin

**Affiliations:** The University of Hong Kong School of Public Health, 7 Sassoon Road, Pokfulam, Hong Kong SAR, China; Geneva Transformative Governance Lab, Global Studies Institute, University of Geneva, Geneva, Switzerland; The University of Hong Kong School of Public Health, 7 Sassoon Road, Pokfulam, Hong Kong SAR, China; The University of Hong Kong School of Public Health, 7 Sassoon Road, Pokfulam, Hong Kong SAR, China; The University of Hong Kong School of Public Health, 7 Sassoon Road, Pokfulam, Hong Kong SAR, China; The University of Hong Kong School of Public Health, 7 Sassoon Road, Pokfulam, Hong Kong SAR, China; State Key Laboratory for Diagnosis & Treatment of Infectious Diseases, the First Affiliated Hospital, School of Medicine, Zhejiang University, Hangzhou, Zhejiang 300013, China; Institute for Social Medicine, Zhejiang University School of Medicine, Hangzhou 310058, China; The University of Hong Kong School of Public Health, 7 Sassoon Road, Pokfulam, Hong Kong SAR, China

**Keywords:** Antimicrobial use, antimicrobial resistance policy, infectious disease prevention, national and sub-national policies, multi-level governance

## Abstract

Against the backdrop of universal healthcare coverage and pre-existing policies on antimicrobial use, China has adopted a state-governed, multi-level, top-down policy governance approach around an antimicrobial resistance (AMR) national action plan (NAP). The Plan relies on tightening control over antimicrobial prescription and use in human and animal sectors. At the same time, medical doctors and veterinarians operate in an environment of high rates of infectious diseases, multi-drug resistance and poor livestock husbandry. In exploring the way that policy responsibilities are distributed, this study aims to describe how Guangdong as a province adopts national AMR policies in a tightly controlled public policy system and an economy with high disparity. We draw on an analysis of 225 AMR-relevant Chinese policy documents at the national and sub-national levels. We adopt a multi-level governance perspective and apply a temporal sequence framework to identify and analyse documents. To identify policy detail, we conducted keyword analysis using the Consolidated Framework for Implementation Research (CFIR) on policies that conserve antimicrobials. We also identify pre-existing medical and public policies associated with AMR. Our findings highlight the emphasis and policies around antimicrobial use regulation to address AMR in China.

**Key messages**

- Against the backdrop of universal healthcare coverage and limited pre-existing policies on antimicrobial use, China has adopted a state-governed, multi-level, top-down policy governance approach around an antimicrobial resistance (AMR) national action plan (NAP).
- The Plan relies on tightening control over antimicrobial prescription and use in human and animal sectors.
- At the same time, medical doctors and veterinarians operate in an environment of high rates of infectious diseases, multi-drug resistance and poor livestock husbandry.
- In exploring the way that policy responsibilities are distributed, this policy content analysis aims to describe how Guangdong as a province adopts national AMR policies in a tightly controlled public policy system and an economy with high disparity.
- Our findings highlight the emphasis on antimicrobial use regulation and its policy approaches. We also investigated pre-existing general medical and public policies with paths for AMR policies.

## Introduction

Antimicrobial resistance (AMR) is considered one of the most difficult global health challenges of the 21st century. China is regarded as one of the nations with high antimicrobial use, high AMR prevalence across human and animal sectors (Pan *et al.*, 2010; Hu *et al.*, 2018) and under-investigated antimicrobial residue and AMR contamination in the environment and food chain (Chen *et al.*, 2010; Yan *et al.*, 2010; Cui *et al.*, 2016; Qiao *et al.*, 2018). Regardless of the microbes’ origin, AMR already has had broad societal impact. The burden of AMR on society is especially prominent in countries with developing economies, high economic disparity and social inequality (Vernet *et al.*, 2014). Recent research by the World Bank indicates that AMR will elevate the rate of poverty and will disproportionally affect low-income countries and vulnerable economies (Dadgostar, 2019). Resistant antimicrobial transmission causes severe or untreatable infections among the human and animal populations and creates a vicious cycle for those living with no or low access to good hygiene or medical care (Nadimpalli *et al.*, 2020).

A key issue in addressing AMR is what actions or policies countries undertake to prevent or mitigate the problem. Following the adoption of a WHO Global Action Plan (GAP) in 2015, countries committed to developing their own national action plans (NAPs). By 2021, 73 of 194 member states had self-reported NAP formulation and 113 of 194 member states had signed the ‘Call to Action’ on AMR (Bhatia, 2020) (www.who.int). This nearly 39 to 58% uptake suggests different degrees of national commitment to prioritize policy attention or sustain political championship. In addition to WHO’s call for the One Health approach, AMR strategies are adopted by the World Organisation for Animal Health (OIE), the Food and Agriculture Organization (FAO) and the United Nations Environment Programme (UNEP). Yet concerning these policy responsibilities, there exist large gaps in our understanding of policy prioritization across different sectors and levels of governance.

Another challenge is to adopt relevant AMR strategies at the national and sub-national levels. Sub-national implementation challenges are observed even when NAPs align with the GAP (Munkholm and Rubin, 2020). Countries with developing economies are especially vulnerable to a lack of resources to evaluate and identify appropriate policy strategies at all levels of office and in all sectors. For instance, it is difficult to earmark resources, strengthen antimicrobial governance systems or set up surveillance systems that reach sub-national states in large countries such as India (Bhatia and Walia, 2017). On the contrary, in Tanzania, the NAP works to promulgate policy sub-nationally. Mdegela *et al.* indicated that the Tanzanian NAP has led to improved coordination of the committee for implementation of activities, better community awareness, development of surveillance sites and successful guideline establishment (Mdegela *et al.*, 2021). Although recent data indicate that China has been able to reduce its antimicrobial use (Xiao *et al.*, 2020b), little is known on how its policy handles conflict of interest and the demand on governance and infrastructures particular to antimicrobial use reduction (Li *et al.*, 2012). In the food-producing animal sector, how China’s policies become adopted as use-scenario or antimicrobial-class control also deserves further exploration (Orubu *et al.*, 2020). The nuanced detail and temporal sequence of China AMR policies underlines the approach, distribution and tempo of its policy implementation.

The goal of this article is to describe how the Chinese province of Guangdong adopts national AMR policies in a tightly controlled public policy system and an economy with high disparity. To better understand the policy distribution, tempo and approaches of China’s antimicrobial use policies, the authors conducted a qualitative analysis of policies planned and implemented in Guangdong, China. Furthermore, a detailed and systematic analysis of China’s AMR policies was conducted to understand how a rapidly developing country and one of its most populous provinces reduce antimicrobial use. Resembling other public policies in China, AMR policies are promulgated through ‘state governance’, that is ‘the process through which the highest authority of the state controls and manages society by administrative, legislative and judicial organs and the decentralization between states and local authorities’ (Bai and Liu, 2020).

To systematically identify details in policy implementation, AMR keywords were identified using the AMR-PACT and AMR-Intervene variables (Ogyu *et al.*, 2020b; Léger *et al.*, 2021). These keywords were then categorized within the Consolidated Framework for Implementation Research (CFIR) for thematic analysis (Keith *et al.*, 2017). In our subsequent analysis, we then focused on three policy questions: (1) Prior and subsequent to WHO’s AMR Global Action Plan (GAP), what were/are the AMR policy emphases in China’s national policies and Guangdong’s sub-national policies; (2) How are responsibilities distributed between the policies of Guangdong province and those of China; and (3) What is the temporal sequence of these policies in human and food-producing animal sectors?

## Methods and materials

### Literature identification

**Figure 1.**
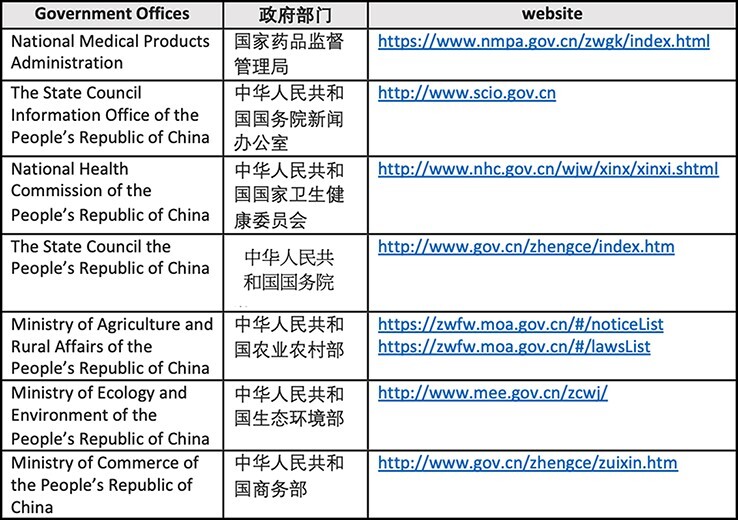
Government websites used for document search

We compiled a grey literature policy document dataset to investigate Chinese national and Guangdong sub-national AMR policies according to pre-content search and compilation, and conducted quantitative and qualitative analysis as indicated in Supplementary Appendix 1. The research team applied the READ approach to systematically identify and examine the policy documents written in Chinese (Dalglish *et al.*, 2020). The team also applied the keyword as the syntactical unit of analysis for reliable variable identification (Krippendorff, 2018). National policies were identified on the Chinese Central Government’s official administrative web portal in the human, animal, food and environmental sectors (Figure 1). The local Chinese search engine ‘Baidu’ was added to locate published grey literature. Sources and documents were verified with Chinese AMR policy researchers and academicians.

The keywords to identify the policies were derived from the AMR GAP, China NAP, AMR-PACT and AMR-Intervene (Ogyu *et al.*, 2020b; Léger *et al.*, 2021). The keywords were translated into Chinese for the search. The content, syntax and titles of the documents included in the database were: (1) relevant to antimicrobial use and AMR policies in the language of Chinese and/or English; (2) published between 1999 and 2020; and (3) derived from official, working and implementation documents at the Chinese national, Guangdong provincial and Guangdong city levels. The data extracted included policy directives, the levels of administrative bureaucracy involved and policy approaches. Document review was considered complete at the point when further iteration of the keywords derived no new policy documents. Policies and variables were reverse-translated to English for analysis. Translation validity was checked by direct and reverse check, as well as contextual verification.

### Thematic analysis and categorization of national and sectoral policies

China’s national AMR policies promulgated from the country’s NAP were defined as policies adopted by two Constituent Departments of the State Council and one State Administration under the State Council: the National Health Commission, the Ministry of Agriculture and Rural Affairs and the National Medical Products Administration (the Chinese Food and Drug Administration prior to 2018) under State Administration for Market Regulation (Figure 1). Sub-national-level documents were defined as those that included relevant keywords in the title and/or content of the document and pertained to the province of ‘Guangdong’, or to its corresponding prefectures, counties, townships and villages in Zengcheng, Zhuhai, Zhongshan and Shantou.

Policy documents were categorized by title and content according to AMR policy categorization variables in AMR-PACT and AMR-Intervene frameworks for their relevance to AMR policy analysis (Léger *et al.*, 2020; Ogyu *et al.*, 2020a). At the sector level, policy documents were categorized according to the sector’s target populations and settings. For the human sector, categorization keywords included, but were not limited to, ‘hospitals’, ‘community pharmacy’, ‘clinics’, ‘patients’, ‘medical doctors’ and ‘laboratory staff’. For the animal sector, categorization keywords included, but were not limited to, ‘farms’, ‘animal feed’, ‘veterinary drugs’, ‘vet drug pharmacopeia’, ‘farmers’ and ‘veterinarians’.

### Qualitative analysis

Keywords from policy title and content were documented and categorized as variables entered into an Excel worksheet. These variables included, but were not limited to, ‘antimicrobial stewardship’, ‘distribution and sales’, ‘antimicrobial production’, ‘diagnostics’, ‘pharmacy’ and ‘internet sales’. These keywords were then categorized and analysed with reference to the CFIR framework (Seale and Tonkiss, 2012; Keith *et al.*, 2017), with emphases on implementation science for effective policy intervention (Damschroder *et al.*, 2009). The CFIR domains included policy intervention characteristics, features, inner and outer settings and policy process. Outer settings described national, provincial, city or village levels. For policy process, all policy documents included were at the implementation phase. The varying variables including intervention characteristics, features and inner settings were categorized in Supplementary Appendices 2, 3 and 4.

### Temporal categorization

The policy documents were arranged in chronological order according to the year of publication. Two researchers, using an Excel database, independently sorted the policy documents and then verified them for reliability and validity.

## Material

**Figure 2.**
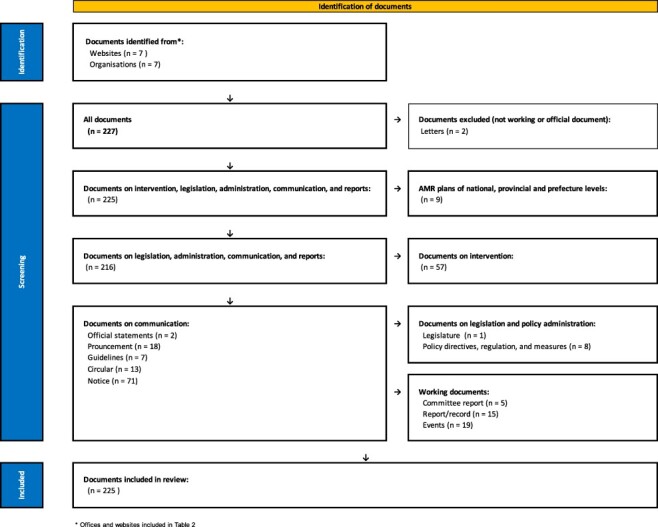
Identification, screening and inclusion of policy documents

Documents related to AMR policy implementation were identified from seven Chinese Government official websites. A total of 227 policy documents were identified; two were excluded and 225 were included. Figure 2 illustrates the document categories. Of the 225 documents included, 205/225 (91%) were official documents including policy directives, national, provincial and sub-provincial plans, official statements, pronouncements and Notices. The remaining were working documents (20/225, 9%), including committee reports and records.

## Results

Analyses of policy documents yield three main findings. First, AMR policies in China are distributed across national and sub-national levels, across settings and social groups and across human, food and animal sectors. Second, these policies vary by policy characteristics, features and settings. Third, pre-existing governance, the public and medical policies play important roles in how and what AMR policies are established and implemented over time.

### Antimicrobial policy implementation distribution in Guangdong, China

**Figure 3.**
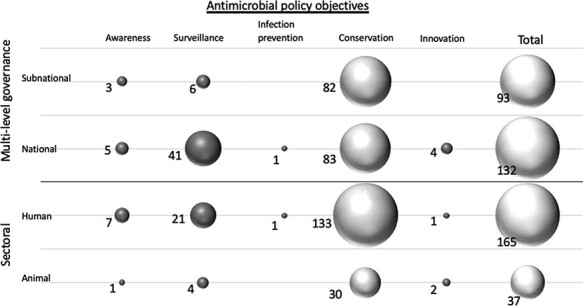
AMR policy adopted in human and animal sectors in China, Guangdong province (1999–2020)

**Figure 4.**
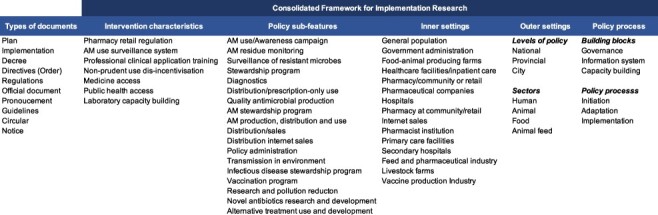
AMR policy implementation keywords in the Consolidated Framework for Implementation Research (CFIR)

Altogether, 165 of the 225 (165/225, 73%) Chinese AMR policy documents focused on the reduction of antimicrobial use (Figure 3). According to CFIR (Figure 4), the antimicrobial conservation policies involved multiple policy characteristics, features and settings that were distributed between national and sub-national (provincial, city and village) levels. Among the Guangdong policies, most targeted settings were teaching and tertiary hospitals where policies focus on restriction of third- and fourth-generation antimicrobial use. Among policies adopted in the human sector, one set of policies built administrative systems at national (41/225, 18%) and Guangdong provincial levels (6/225, 3%) (Figure 3). In primary and secondary hospitals, fundamental policies aimed to build capacity for antimicrobial clinical prescription and AMR laboratory diagnostics. Policy information system infrastructure building was also observed in Guangdong prefecture-level city hospitals’ ‘Sunshine Medical System’ (Supplementary Appendix 2.1), which engaged clinical pharmacists and an antimicrobial use audit. About 22 of the 225 (10%) policy documents aimed to regulate antimicrobial manufacturing, quality control, drug sales and distribution.

The second policy focus was on the AMR surveillance system. Policies with this focus were mostly identified at the national level (132/225, 59%) and in the human health sector (163/225, 72%) (Figure 3). Notices were published regarding the surveillance of the rational use of antimicrobials and the scientific management of AMR. To effectively implement a surveillance system, policies which intended to strengthen hospitals’ information reporting and clinical diagnostics training were heavily adopted. These policies placed a strong focus on national standards and on enhancing diagnosis capacities. A further two policies were established to improve bacterial and fungal infection diagnosis (Supplementary Appendix 2.2). Training courses were implemented to ensure good practice on bacterial resistance diagnosis and reporting. In the same year, the establishment of a monitoring network was announced (Supplementary Appendices 2.3 and 2.4). These interventions formed the basis for China’s AMR and use surveillance systems. Subsequent Notices on rational drug use and their monitoring were re-iterated in Guangdong province (Supplementary Appendices 2.5 and 2.6).

### Prioritizing antimicrobial use reduction policies

Of the various AMR policy intentions, China prioritized antimicrobial use regulation and demonstrated a reducing trend in use (Xiao *et al.*, 2020a). The regulation is also considered one of the harder policies to implement (Schoenmakers, 2020). To that end, there are three main characteristics in China’s approach. First, AMR policies were implemented around pre-existing policies and the merits of existing infrastructure and governance characteristics. Second, dis-incentivization policy instruments were applied in medical service systems. Third, various types of policy instruments were promulgated to complement and synergize regulatory policies. These policies combined responsibility delegation and education, professional training, antimicrobial manufacture quality control, antimicrobial sales and distribution regulation and laboratory diagnostics enhancement.

The National Essential Medicines List (2009), along with the Zero-Mark Up and National Health Insurance (2011) policies, provided the infrastructure for national health, medical services and eventually antimicrobial prescription regulation (Supplementary Appendices 2.7 and 2.8). Additionally, the National Pharmaceutical Classification and Management (2005) policy supplemented this infrastructure with a demarcation between prescription and non-prescription drugs. Three additional regulations on pharmaceutical administration were formulated in 2011 which collectively set the administration foundation for antimicrobial use regulations (Supplementary Appendices 3.1 and 3.2). These infrastructure and governance systems in turn provided points of access to antimicrobial prescription training and prescription management interventions in Guangdong and sub-provincial hospitals (clinical antimicrobial use Notices in 2008, 2009 and 2012). Such on-the-job training transferred knowledge of antimicrobial use and secured buy-in among medical practitioners. The training and infrastructure also helped build capacity to delink sales-oriented financing in hospitals and shift to infection diagnostics and treatment services (Supplementary Appendix 2.9) (Wang *et al.*, 2016). Together with the Essential Medicines List and the drug registry, the Universal Health Insurance system provided the hospital management infrastructure at various state levels. These infrastructure and governance systems laid the foundation to restrict the prescription of critically important antibiotics in tertiary and secondary hospitals. In subsequent years, a national campaign on prudent antibiotic use was promulgated among these policy foundations.

Statutes 48 and 84 were two regulatory antimicrobial use policies that enforced dis-incentivization of non-prudent antimicrobial use. In 2006, the Health Bureau published Statute 48 which provided specific regulation targeted at prophylactic antimicrobial use and critically important antimicrobials (Supplementary Appendix 2.10). This Statute featured clinical application training, established governance on clinical use by responsibility delegation among hospital and office leaders and offered a clarified pharmaceutical list regarding critical antibiotics. The second clause of the Statute restricted fluoroquinolone use to culture-sensitivity laboratory result verification, and banned it from routine use in gastrointestinal and urinary-system diseases, as well as in urinary surgery prophylactic use. In the same year, further antimicrobial stewardship training programmes and activities (Notices 731, 32, 37, 288 and 40) were initiated in the Guangdong hospitals. These programmes focused on clinical antimicrobial use guidelines, hospital pharmaceutical management and a multi-agency antibacterial control plan in townships and cities (Notices 285, 38, 161, 28, 111 and 56). In 2012, Decree 84 was published in addition to several Ministry of Health Orders (Supplementary Appendix 2.11). This Decree was a major gesture of administrative commitment to reduce antimicrobial prescription in hospitals at the provincial, township and village levels. It included penem-class and tigecycline antimicrobials regulatory rules and use audit. Clauses 46 and 50 to 55 of the Decree underscored the various forms of dis-incentivization, including financial penalty, demotion, de-registration and release from position for medical practitioners who obtained financial gain from antimicrobial prescription, over-prescription or non-prudent prescription.

Several policy interventions were implemented to support regulatory policies. These instruments included responsibility delegation, governance clarification, office coordination and professional training to change antimicrobial prescription behaviour. For instance, clinical pharmacists were given governance roles in hospitals and communities to restrict antimicrobial prescription. To implement this policy, the national pharmacists’ examination was revised to test knowledge of regulations, laws and clinical regarding antimicrobials, as well as their application (2013). Subsequently, pharmaceutical classification regulation and pharmacists training were implemented in Guangdong province in 2016. Nationally, in order to build infrastructure and plan governance around antimicrobial use control, inter-office collaboration was implemented. Consequently, the Ministry of Health, the State Food and Drug Administration, the Ministry of Industry and Information Technology and the Ministry of Agriculture jointly issued a collaborative joint plan to regulate antimicrobial use in the human, animal and food sectors (Supplementary Appendix 2.12). Other policies which aimed to strengthen education and professional training—‘Microbial ONLINE’ and ‘Peiwei ONLINE’ programmes—were established to improve clinical microbiologists’ ability to diagnose bacterial and fungal infections. Furthermore, the national antimicrobial prescription policy laid down basic antimicrobial use technical information on bio-availability, route of administration, antimicrobial choice, dosage and duration of treatment (Supplementary Appendix 2.13). In the same year, the Guangdong Department of Health implemented antimicrobial clinical use guidelines on critically important antibiotics (third- and higher generation cephalosporins) and attached restrictions on prescriptions according to the medical doctor’s seniority.

The Guangdong Ministry of Health, Agriculture and Rural Affairs, and the Food and Drug Administration also implemented policies to improve pharmaceutical manufacture quality control and pharmaceutical sales tracking in the human and food-animal sectors. The approach works on the premise that legitimate pharmaceutical quality reinforces clinicians’ and patients’ confidence to comply with antimicrobial choice and course to reduce antimicrobial escalation or over-use (Delepierre *et al.*, 2012; Pan *et al.*, 2016). To regulate the quality of drug manufacturing, the Ministry of Health implemented a policy on manufacture governance and monitoring in the pharmaceutical sector (Supplementary Appendix 3.3). By the Statute, the Food and Drug Administration also investigated and reprimanded illegal sales of antibacterial drugs (Supplementary Appendix 3.4). To regulate antimicrobial use in livestock, policies to control distribution and retail were documented (Supplementary Appendix 4.1). To establish an information system for antimicrobial sales regulation, a monitoring infrastructure was implemented in Guangdong (Supplementary Appendix 4.2). This infrastructure included QR-code tracking labelling on antimicrobial containers for food-producing animals.

### Temporal sequence of antimicrobial resistance policies in Guangdong, China

**Figure 5.**
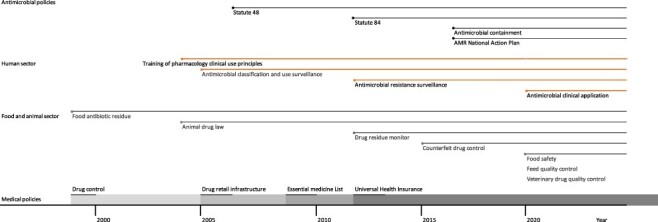
Pooled antimicrobial conservation policies illustrated by time-series in human and food-producing animal sector

The Drug Control Ordinance was first established by the Department of Health in 1984 to regulate general drug production, sales, distribution and prescription in the health service sector. In the subsequent 13 years until 1997, at least 12 national laws and regulations were introduced on general drugs and pharmaceutical regulation. Eight of these aimed to regulate non-prudent antimicrobial use in hospitals. Four other Statutes sought to regulate antimicrobial retail sales and distribution, agency collaboration, governmental infrastructure building and antimicrobial manufacturing by-product disposal at the sub-national levels (Figure 5). These policies served to build foundations and initiated capacity building for infrastructure and governance of antimicrobial use regulation at the provincial, township and village levels.

**Figure 6.**
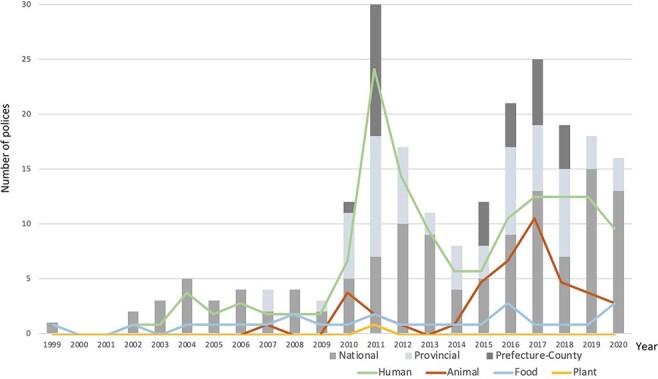
Histogram on distribution of AMR policies between 1999 and 2020 categorized by national-, provincial- and prefecture-level city or county. Lines depict multi-sectoral distribution of policies

Antimicrobial use regulation policies were established over 20 years, with some intensity in the year 2011 (Figure 6). Between 2005 and 2009, the National Ministry of Health and the State Administration of Chinese Medicine established policies that targeted the training of clinical antimicrobial use. In 2005, these two Ministries and the Ministry of Health of the General Logistics Department jointly established a monitoring network for the clinical application of antibiotics and bacterial drug resistance. In the same year, Guangdong issued a Notice on the regulation of antimicrobial clinical use (Supplementary Appendix 2.14). Between 2007 and 2010, the city of Guangzhou implemented regulation governance within the Sunshine System (Supplementary Appendix 2.15). Another set of antimicrobial use regulation policies was established in 2011. More than six AMR-related policies were implemented in Guangdong province and its prefecture-level cities in Shantou, Zhongshan and Zhuhai. The Notices covered clinical use outcome monitoring, training intervention and antimicrobial use surveillance implementation.

One of the major antimicrobial policy characteristics was the development of policies with different policy features which accumulated to disincentivize policies. In 2006, policy Statute 48 emphasized hospital infection control and antimicrobial use control. In 2012, Statute 84 led the General Office of the National Health and Family Planning Commission to implement multiple interventions regarding hospital infection management in Primary Care Facilities in Guangdong (No. 40, Medical Affairs Office of the State Health Bureau) (Figure 5). These antimicrobial regulation policies adopted five policy sub-features: training in clinical application of antibacterial drugs, cataloguing and classification of antimicrobials (2012 edition), drug administration, hospital infection management and establishment of an antibacterial drug monitoring system. After the implementation of Statute 84 at the national level, Guangdong implemented regulation on pharmaceutical retail in 2014 and its city of Rongcheng implemented an inspection system on the regulation of pharmaceutical prescription (Supplementary Appendix 2.16). In 2015, a national follow-up was conducted on the regulation of prudent antimicrobial use and research to support stewardship policies (Supplementary Appendix 2.17). In 2016, Guangdong continued to implement measures to supervise and administer drug transactions and constructed a direct antimicrobial use report system (Supplementary Appendix 2.18).

China’s AMR NAP led to a number of policy interventions in medical services and pharmaceutical administration in 2016 and beyond. In hospitals, AMR containment guidelines and intervention were implemented against NDM-1 pan-drug-resistant enterobacteriaceae infections (Figure 5). Training programmes for diagnosis and treatment of bacterial and fungal infection were also established. In pharmaceutical administration, the Food and Drug Production and Operation Risk Classification Management led the Office of Guangzhou Food and Drug Administration to issue a three-year plan on the supervision and administration of prescription pharmaceuticals, the standardization of medicine use and professional education on prescription (Supplementary Appendix 3.5). Combining antimicrobial clinical use and sales regulation, the Special Committee for the Management of Clinical Rational Use of Pharmacology of Guangdong Province held training programmes on prudent antimicrobial use and general pharmaceutical safety, antimicrobial management and clinical monitoring network technical application (Supplementary Appendix 2.19). Additionally, the State Drug Administration issued a Notice to regulate the licensure of pharmacists and their roles and responsibilities in observing pharmaceutical retail regulation (Supplementary Appendix 3.6).

Earlier Statutes helped facilitate the formulation of targeted policies against feed additives and categorized banned drugs in food-producing animals. The earlier antimicrobial-relevant policies formulated in the animal sector in China aimed solely at food-safety regulations proxied by antibiotic residue in food commodities. The Department of Agriculture announced three laws concerning the food-producing animal sector between 1994 and 1999 (Figure 5). These policies did not target the banning or reduction of non-prudent antimicrobial use in livestock or food-producing animals. Rather, the first policy document on antimicrobial use in animals was formulated as part of the regulation of fertilisers, anti-parasitics and antibiotics that are unsafe for human consumption. In 2001, antimicrobial residue regulations began to emerge at the national level. In 2004, the State Council established new national policies on veterinary drug administrative approaches to control and regulate all pharmaceuticals used in animal farming in China (Supplementary Appendix 4.3). The AMR-directed policy on raising food-producing animals was identified in a document published in 2015. It was designed to regulate antimicrobials in pre-mixed feed, in-feed mass medication and individual antimicrobial treatments. Before the animal AMR NAP was formulated, two Statutes were devised to regulate by pharmaceutical additives in animal feed, veterinary prescriptive pharmaceuticals and over-the-counter pharmacecutical sales in livestock farming (Supplementary Appendix 4.4). After the NAP was adopted, more than four measures were implemented in veterinary pharmaceutical registration and administration (Supplementary Appendix 4.5). Subsequently, several regulatory policies were implemented on the veterinary pharmaceuticals flomefloxacin, pefloxacin, ofloxacin, norfloxacin, pre-mixed quinoxaline, arsanilic acid, roxarsone, certain livestock and aquatic antibiotics and veterinary drug residues. Counterfeit antimicrobial ban and antimicrobial tracking policies were implemented and targeted at pharmaceutical and farms in Guangdong and its county Guangxi. The Ministry of Agriculture specified further tightening of regulations on pharmaceutical registration, quality control in manufacturing and additives in animal feed in Guangdong province and its farming villages (Supplementary Appendix 4.6). In particular, the use of chloramphenicol, vancomycin and colistin in food-producing animals was banned. In 2020, the implementation of national feed quality check plans, milk quality and safety monitoring and a ban on antimicrobials as livestock growth promoters reflected the development of antimicrobial regulatory policies in the food-producing animal sector (Supplementary Appendix 4.7).

## Discussion

We use Guangdong province as a case study to explore the time scale, complexity and development of AMR policy adoption and implementation. One of the limitations of this study is a lack of documents when national, provincial, prefectural, city or village AMR policies are not published on official websites or Baidu. Even with this limitation, a few observations can be made about AMR policy adoption in China. The layering, waves and temporal sequence of the policies in Guangdong reveal some of the policy approaches and conditions for AMR policy implementation. General policies such as universal medical services, pharmaceutical governance, professional education and administrative public policies need to be in place when and where targeted antimicrobial policies are to be implemented. In many countries, especially the low- to middle-income countries, such background policies, infrastructure and governance are often suboptimal for antimicrobial use reduction policy implementation. In these situations, general public and medical service policies formed at the national or local level should be identified, or formulated, to enhance medical services and implement antimicrobial policies.

Antimicrobial use reduction is one of the more difficult policies to implement and sustain. In Pakistan, the lack of powerful stakeholder commitment is a shortcoming for antimicrobial use policy implementation (Khan *et al.*, 2020). In the UK, such a policy has to work through heavy lobbying for deregulation or public subsidy (Glover *et al.*, 2020). One clear observation that emerges from the analyses of documents in Guangdong, China, is how the stakes and responsibilities are delegated and distributed among targeted settings and stakeholders at the national, provincial and rural village levels. Precisely because such policies need to be adopted in layers according to policy infrastructure and governance infrastructure, China weaves governance features into its AMR policies, which bears similarity to Thailand’s AMR NAP sixth strategy on ‘Governance Mechanism’. Such consideration for governance is significant for China and Thailand’s AMR policy implementation and likely the same for other countries that are developing and adopting AMR policies alongside evolving health and public health policy systems (Birgand *et al.*, 2018; Legido-Quigley *et al.*, 2018; Chua *et al.*, 2021; Frumence *et al.*, 2021; Yin *et al.*, 2021). Indeed, the coupling of governance and administrative capacities with technical policies is perhaps one of the contextual drivers for sustaining AMR policy implementation (Birgand *et al.*, 2018; Yin *et al.*, 2021).

The temporal sequence of Chinese AMR-related and general health policies (Parsons *et al.*, 2019) shows that pre-existing policies can help scaffold and support AMR policy implementation. For instance, prior policy adoption related to healthcare services including the Zero Mark-Up and Essential Medicine Policies plays a significant role in AMR policy implementation in China. Compared to other countries such as India, where the pharmaceutical industry has a dual role in sponsoring medical practitioner training and self-monitoring antimicrobial sales, the Zero-Mark Up policy in China can gatekeep and limit conflict of interest and disincentivize antimicrobial over-prescription. However, such a policy is not without criticism, especially as it poses challenges to hospital incomes (Hiilamo and Glantz, 2015; Kessy, 2018). To overcome the problem of pharmacy budget dominance (Vickers *et al.*, 2019), reform was launched in 2009 by the Chinese State Council Statute No. 12 which aimed to increase hospital budgets through government subsidies and service fees. In 2011, by Order No. 674, the Chinese National Development and Reform Commission implemented disease-based and diagnostic-related group-based payment (Xu *et al.*, 2019). This provided governance and infrastructure for antimicrobial prescription stewardship, with the implementation of capitation pay-for-performance implemented in 2012 (Shen *et al.*, 2022). Between 2015 and 2017, the Chinese State Council established health policy Statutes Nos. 12, 33, 38, 70 and 55, which led to the establishment of the National Healthcare Security Administration established in 2018 to manage the financing of pharmaceutical and medical services financing in public hospitals.

To strengthen AMR policy governance experiences, China can refer to a number of countries, including Tanzania, for insights on multi-sectoral coordination and AMR policy evaluation, feedback and reporting system challenges (Frumence *et al.*, 2021). China can partner with international advocacy to engage national policy-makers (Anderson *et al.*, 2019) as well as support global treaties to improve implementation transparency and accountability (Ruckert *et al.*, 2020). Future AMR policies in China may involve agency, AMR policy and programme evaluation similar to that of the UK’s AMR NAP (2019–2024). China may also involve pharmaceutical public–private partnerships similar to that of the UK–US ‘Combating Antibiotic Resistant Bacteria Biopharmaceutical Accelerator (CARB-X)’ (2016). For China’s laboratory capacity building, its microbiologists can continue to partner with the Global Antimicrobial Lab & Response Network Collaboration (Shu *et al.*, 2019). AMR policies in the food sector in China have referred to standards stated in the Codex Alimentarius (initiated in 2006 by the Food and Agriculture Organization) and endorsed by the AMR Tripartite Alliance (World Organization for Animal Health (OIE), Food and Agriculture Organization (FAO) and World Health Organization (WHO)). In addition to China’s regulation of food antimicrobial residue, further policies and programmes to train veterinarians on appropriate antimicrobial use in livestock, and farm managers on appropriate husbandry in farm production, as well as to improve the performance of veterinary services (PVS) are important. All in all, to solve AMR issues globally and locally, further investigation of AMR policy distribution, tempo and context in different countries and their sub-national situations can help strengthen policy implementation.

## Supplementary Material

czac052_Supp

## Reflexivity statement

The authors include four female and five male authors and span multiple levels of seniority. Our authors include four AMR policy specialists from North America, Europe and Asia. In addition to health economists, microbiologists and epidemiologists, authors have extensive experience in qualitative research, public education and clinical fieldwork in China.

## References

[R1] Anderson M , Schulze K, Cassini A, Plachouras D, Mossialos E. 2019. A governance framework for development and assessment of national action plans on antimicrobial resistance. *The Lancet Infectious Diseases* 19: e371–84. 31588040 10.1016/S1473-3099(19)30415-3

[R2] Bai Z , Liu J. 2020. China’s governance model and system in transition. *Journal of Contemporary East Asia Studies* 9: 65–82.

[R3] Bhatia R . 2020. ABC of antimicrobial resistance control. *Journal of Public Health Policy* 41: 225–7. 31965047 10.1057/s41271-019-00215-z

[R4] Bhatia R , Walia K. 2017. Combating antimicrobial resistance in India: technical challenges & opportunities. *Indian Journal of Medical Research* 146: 683–7. 29664025 10.4103/ijmr.IJMR_19_17 PMC5926338

[R5] Birgand G , Castro-Sánchez E, Hansen S et al. 2018. Comparison of governance approaches for the control of antimicrobial resistance: analysis of three European countries. *Antimicrobial Resistance and Infection Control* 7: 1–12. 29468055 10.1186/s13756-018-0321-5 PMC5819189

[R6] Chen X , Naren G-W, Cong-Ming W et al. 2010. Prevalence and antimicrobial resistance of Campylobacter isolates in broilers from China. *Veterinary Microbiology* 144: 133–9. 20116182 10.1016/j.vetmic.2009.12.035

[R7] Chua AQ , Verma M, Hsu LY, Legido-Quigley H. 2021. An analysis of national action plans on antimicrobial resistance in Southeast Asia using a governance framework approach. *The Lancet Regional Health-Western Pacific* 7: 100084. 10.1016/j.lanwpc.2020.100084 PMC8315476 34327414

[R8] Cui M , Xie M, Zhina Q et al. 2016. Prevalence and antimicrobial resistance of Salmonella isolated from an integrated broiler chicken supply chain in Qingdao, China. *Food Control* 62: 270–6.

[R9] Dadgostar P . 2019. Antimicrobial resistance: implications and costs. *Infection and Drug Resistance* 12: 3903–10. 31908502 10.2147/IDR.S234610 PMC6929930

[R10] Dalglish SL , Khalid H, McMahon SA. 2020. Document analysis in health policy research: the READ approach. *Health Policy and Planning* 35: 1424–31. 10.1093/heapol/czaa064 PMC7886435 33175972

[R11] Damschroder LJ , Aron DC, Keith RE et al. 2009. Fostering implementation of health services research findings into practice: a consolidated framework for advancing implementation science. *Implementation Science* 4: 1–15. 19664226 10.1186/1748-5908-4-50 PMC2736161

[R12] Delepierre A , Gayot A, Carpentier A. 2012. Update on counterfeit antibiotics worldwide; public health risks. *Medecine et Maladies Infectieuses* 42: 247–55. 22621827 10.1016/j.medmal.2012.04.007

[R13] Frumence G , Mboera LEG, Sindato C et al. 2021. The governance and implementation of the National Action Plan on Antimicrobial Resistance in Tanzania: a qualitative study. *Antibiotics* 10: 273. 10.3390/antibiotics10030273 PMC7998560 33803077

[R14] Glover RE , Mays NB, Petticrew MP, Thompson C. 2020. OP59 stakeholder narratives of ‘problems’ and ‘solutions’: analysing the 2018 Health and social care committee antimicrobial resistance submissions in the United Kingdom. BMJ Publishing Group Ltd.

[R15] Hiilamo H , Glantz SA. 2015. Implementation of effective cigarette health warning labels among low and middle income countries: state capacity, path-dependency and tobacco industry activity. *Social Science & Medicine* 124: 241–5. 25462428 10.1016/j.socscimed.2014.11.054 PMC4276461

[R16] Hu F , Zhu D, Wang F, Wang M. 2018. Current status and trends of antibacterial resistance in China. *Clinical Infectious Diseases* 67: S128–34. 30423045 10.1093/cid/ciy657

[R17] Keith RE , Crosson JC, O’Malley AS, Cromp D, Taylor EF. 2017. Using the Consolidated Framework for Implementation Research (CFIR) to produce actionable findings: a rapid-cycle evaluation approach to improving implementation. *Implementation Science* 12: 1–12. 28187747 10.1186/s13012-017-0550-7 PMC5303301

[R18] Kessy AT . 2018. Decentralisation, local governance and path dependency theory. *Utafiti* 13: 54–76.

[R19] Khan MS , Durrance-Bagale A, Mateus A et al. 2020. What are the barriers to implementing national antimicrobial resistance action plans? A novel mixed-methods policy analysis in Pakistan. *Health Policy and Planning* 35: 973–82. 32743655 10.1093/heapol/czaa065

[R20] Krippendorff K . 2018. *Content Analysis: An Introduction to Its Methodology*. Analytical/Representational Techniques. Sage Publications, pp. 196–214.

[R21] Léger A , Lambraki I, Graells T et al. 2021. AMR-Intervene: a social–ecological framework to capture the diversity of actions to tackle antimicrobial resistance from a One Health perspective. *Journal of Antimicrobial Chemotherapy* 76: 1–21. 33057678 10.1093/jac/dkab197 PMC8314113

[R22] Léger A , Lambraki I, Graells T et al. 2020. AMR-Intervene: a social–ecological framework to capture the diversity of actions to tackle antimicrobial resistance from a One Health perspective. *Journal of Antimicrobial Chemotherapy* 76: 1–21. 10.1093/jac/dkab197 PMC8314113 33057678

[R23] Legido-Quigley H , Khan MS, Durrance-Bagale A, Hanefeld J. 2018. Something borrowed, something new: a governance and social construction framework to investigate power relations and responses of diverse stakeholders to policies addressing antimicrobial resistance. *Antibiotics* 8: 3. 10.3390/antibiotics8010003 PMC6466563 30586853

[R24] Li Y , Jing X, Wang F et al. 2012. Overprescribing in China, driven by financial incentives, results in very high use of antibiotics, injections, and corticosteroids. *Health Affairs* 31: 1075–82. 22566449 10.1377/hlthaff.2010.0965

[R25] Mdegela RH , Mwakapeje ER, Rubegwa B et al. 2021. Antimicrobial use, residues, resistance and governance in the food and agriculture sectors, Tanzania. *Antibiotics (Basel)* 10: 454. 10.3390/antibiotics10040454 PMC8073917 33923689

[R26] Munkholm L , Rubin O. 2020. The global governance of antimicrobial resistance: a cross-country study of alignment between the global action plan and national action plans. *Globalization and Health* 16: 109. 10.1186/s12992-020-00639-3 PMC7656753 33176810

[R27] Nadimpalli ML , Marks SJ, Montealegre MC et al. 2020. Urban informal settlements as hotspots of antimicrobial resistance and the need to curb environmental transmission. *Nature Microbiology* 5: 787–95. 10.1038/s41564-020-0722-0 32467623

[R28] Ogyu A , Chan O, Jasper Littmann J et al. 2020b. National action to combat AMR: a One-Health approach to assess policy priorities in action plans. *BMJ Global Health* 5: e002427.doi: 10.1136/bmjgh-2020-002427 10.1136/bmjgh-2020-002427 PMC7359186 32665430

[R29] Ogyu A , Chan O, Littmann J et al. 2020a. National action to combat AMR: a One-Health approach to assess policy priorities in action plans. *BMJ Global Health* 5: e002427. 10.1136/bmjgh-2020-002427 PMC7359186 32665430

[R30] Orubu E , Samuel F, Sutradhar I, Zaman MH, Wirtz VJ. 2020. Benchmarking national action plans on antimicrobial resistance in eight selected LMICs: focus on the veterinary sector strategies. *Journal of Global Health* 10. 10.7189/jogh.10.020414 PMC7568929 33110576

[R31] Pan H , Luo H, Chen S, Ba-Thein W. 2016. Pharmacopoeial quality of antimicrobial drugs in southern China. *The Lancet Global Health* 4: e300–02. 27102190 10.1016/S2214-109X(16)00049-8

[R32] Pan ZM , Geng SZ, Zhou YQ et al. 2010. Prevalence and antimicrobial resistance of Salmonella sp. isolated from domestic animals in Eastern China. *Journal of Animal and Veterinary Advances* 9: 2290–4.

[R33] Parsons R , Everingham J-A, Kemp D. 2019. Developing social impact assessment guidelines in a pre-existing policy context. *Impact Assessment and Project Appraisal* 37: 114–23.

[R34] Qiao M , Ying G-G, Singer AC, Zhu Y-G. 2018. Review of antibiotic resistance in China and its environment. *Environment International* 110: 160–72. 29107352 10.1016/j.envint.2017.10.016

[R35] Ruckert A , Fafard P, Hindmarch S et al. 2020. Governing antimicrobial resistance: a narrative review of global governance mechanisms. *Journal of Public Health Policy* 41: 515–28. 32908184 10.1057/s41271-020-00248-9 PMC7479750

[R36] Schoenmakers K . 2020. How China is getting its farmers to kick their antibiotics habit. *Nature* 586: S60.

[R37] Seale C , Tonkiss F. 2012. Content and comparative keyword analysis. *Researching Society and Culture* 459: 478.

[R38] Shen L , Wei X, Jia Yin D et al. 2022. Interventions to optimize the use of antibiotics in China: a scoping review of evidence from humans, animals, and the environment from a One Health perspective. *One Health* 14: 100388. 10.1016/j.onehlt.2022.100388 PMC9171522 35686150

[R39] Shu Y , Song Y, Dayan Wang CM et al. 2019. A ten-year China-US laboratory collaboration: improving response to influenza threats in China and the world, 2004–2014. *BMC Public Health* 19: 520. 10.1186/s12889-019-6776-3 PMC6696701 32326921

[R40] Vernet G , Mary C, Altmann DM et al. 2014. Surveillance for antimicrobial drug resistance in under-resourced countries. *Emerging Infectious Diseases* 20: 434. 10.3201/eid2003.121157 PMC3944851 24564906

[R41] Vickers RJ , Bassetti M, Clancy CJ et al. 2019. Combating resistance while maintaining innovation: the future of antimicrobial stewardship. *Future Microbiology* 14: 1331–41. 31526186 10.2217/fmb-2019-0227

[R42] Wang L , Zhang X, Liang X, Bloom G. 2016. Addressing antimicrobial resistance in China: policy implementation in a complex context. *Globalization and Health* 12: 1–9. 27267876 10.1186/s12992-016-0167-7 PMC4893878

[R43] Xiao Y , Shen P, Zheng B et al. 2020a. Change in antibiotic use in secondary and tertiary hospitals nationwide after a national antimicrobial stewardship campaign was launched in China, 2011–2016: an observational study. *The Journal of Infectious Diseases* 221: S148–55. 32176788 10.1093/infdis/jiz556

[R44] Xiao Y , Shen P, Zheng B et al. 2020b. Change in antibiotic use in secondary and tertiary hospitals nationwide after a national antimicrobial stewardship campaign was launched in China, 2011–2016: an observational study. *The Journal of Infectious Diseases* 221: S148–55. 32176788 10.1093/infdis/jiz556

[R45] Xu J , Jian W, Zhu K, Kwon S, Fang H. 2019. Reforming public hospital financing in China: progress and challenges. *BMJ* 365. 10.1136/bmj.l4015 PMC6598707 31227512

[R46] Yan H , Neogi SB, Ziyao M et al. 2010. Prevalence and characterization of antimicrobial resistance of foodborne Listeria monocytogenes isolates in Hebei province of Northern China, 2005–2007. *International Journal of Food Microbiology* 144: 310–6. 21074885 10.1016/j.ijfoodmicro.2010.10.015

[R47] Yin J , Wang Y, Xueran X et al. 2021. The progress of global antimicrobial resistance governance and its implication to China: A review. *Antibiotics* 10: 1356. 10.3390/antibiotics10111356 PMC8614673 34827294

