# Supplementary material for: Unpacking multi-level governance of antimicrobial resistance policies: the case of Guangdong, China
Source: Health Policy Plan. 2022 Jul 1;37(9):1148–57. doi: 10.1093/heapol/czac052 (PMC9558914; doi:10.1093/heapol/czac052)
Supplement: czac052_Supp [file czac052_supp.zip › 18-5-22-Supplementary.docx]

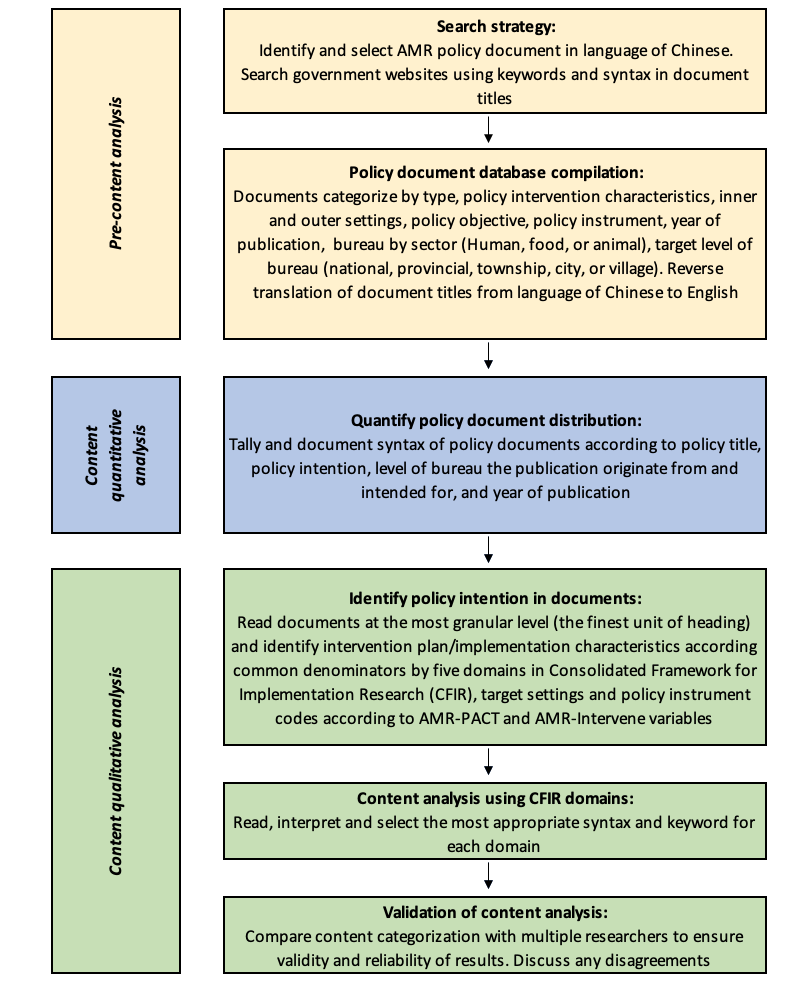


Appendix 1. Pre-content analysis, quantitative, and qualitative keyword analysis

Appendix 2. Key policy features depicted by policy title, characteristics, features, and settings in human healthcare sector

Appendix 3. Key policy features depicted by policy title, characteristics, features, and settings in pharmaceutical sector

Appendix 4. Key policy features depicted by policy title, characteristics, features, and settings in veterinary and livestock sector
